# Risk factors for unplanned reintubation caused by acute airway compromise after general anesthesia: a case-control study

**DOI:** 10.1186/s12871-021-01238-4

**Published:** 2021-01-12

**Authors:** Si Chen, Yuelun Zhang, Lu Che, Le Shen, Yuguang Huang

**Affiliations:** 1 Department of Anesthesiology, Peking Union Medical College Hospital, Chinese Academy of Medical Science & Peking Union Medical College, Beijing, 100730 China; 2 Medical Research Center, Peking Union Medical College Hospital, Chinese Academy of Medical Science & Peking Union Medical College, Beijing, 100730 China

**Keywords:** Airway, Complications, Extubation, General anesthesia, Prognosis

## Abstract

**Background:**

This study aimed to identify the risk factors and evaluate the prognosis of unplanned reintubation caused by acute airway compromise (AAC) after general anesthesia.

**Methods:**

This case-control study included surgical patients who underwent unplanned reintubation in the operating room and postanesthesia care unit after general anesthesia between January 1, 2014, and December 31, 2018. Cases due to AAC were matched 1:4 with randomly selected controls.

**Results:**

A total of 123,068 patients were included, and reintubation due to AAC was performed in 36 patients (approximate incidence 0.03%). Univariable analysis revealed that male sex, age > 65, ASA physical status 3, sepsis, heart disease history, cerebral infarction history, Cormack Lehane grade, surgery type, fresh frozen plasma infusion, increased intubation duration, white blood cell count, and creatinine clearance rate were related to AAC-caused unplanned reintubation. Multivariable analysis revealed that age > 65 (OR = 7.50, 95% CI 2.47–22.81, *P* < 0.001), ASA physical status 3 (OR = 6.51, 95% CI 1.18–35.92, *P* = 0.032), head-neck surgery (OR = 4.94, 95% CI 1.33–18.36, *P* = 0.017) or thoracic surgery (OR = 12.56, 95% CI 2.93–53.90, *P* < 0.001) and a high fluid load (OR = 3.04, 95% CI 1.16–7.99, *P* = 0.024) were associated with AAC-caused unplanned reintubation. AAC-caused unplanned reintubation patients had longer postoperative hospital (OR = 5.26, 95% CI 1.57–8.95, *P* < 0.001) and intensive care unit days (OR = 3.94, 95% CI 1.69–6.18, *P* < 0.001).

**Conclusions:**

Age > 65, ASA physical status 3, head-neck or thoracic surgery and high fluid load were found to be associated with AAC-caused unplanned reintubation.

## Background

Unplanned reintubation refers to intubation after failed extubation. It is a significant adverse event after general anesthesia with tracheal intubation and is often related to postoperative pneumonia, tracheotomy, prolonged stays in the hospital or intensive care unit (ICU), increased hospital costs and mortality [1, 2].

The reasons for unplanned reintubation can vary from a patient’s unstable physical condition [3] to surgical indications, accidental removal of the endotracheal tube and others. Many studies have identified risk factors for reintubation without analysing the causes. Some studies classified the cause of reintubation as airway or non-airway, but most of these studies focused on ICU patients [4, 5]. To our knowledge, no previous study has investigated the risk factors and prognosis of postoperative unplanned reintubation caused by acute airway compromise (AAC) after classifying the cases by cause.

The purpose of this study was to evaluate the risk factors and prognosis of unplanned reintubation caused by AAC in surgical patients after general anesthesia.

## Methods

### Study design and setting

This investigation was a retrospective, case-control study approved by the Peking Union Medical College (PUMC) Hospital Institutional Review Board (S-K745, April 25th, 2019). The requirement for written informed consent was waived by the institutional review board. The basic information of the surgical patients who underwent unplanned reintubation between January 1, 2014, and December 31, 2018, in the PUMC hospital operating room and postanaesthetic care unit (PACU) was obtained from the adverse-event reporting system. All the data related to the patient, anesthesia and operation were collected from the anesthetic recording system and Hospital Information System of PUMC Hospital. This manuscript adheres to the applicable Strengthening the Reporting of Observational studies in Epidemiology (STROBE) guidelines.

### Participants

All surgical cases involving unplanned reintubation in the operating room or PACU from January 1, 2014, to December 31, 2018, were extracted from the adverse-event system and then analysed and categorised by cause. In this study, AAC was defined as a support-required airway situation caused by acute respiratory reasons, such as hypoxia, respiratory muscle fatigue, airway obstruction, residual muscular blockage or phrenic nerve dysfunction. The inclusion criteria were general anesthesia, intubation and extubation in the operating room and non-cardiac operations. Patients who had a preoperative tracheal tube or who remained intubated after the operation were excluded. The AAC-caused unplanned reintubation cases were 1:4 matched with control cases, which were randomly selected from the same database using the same selection criteria.

### Potential risk factors

As stated previously, only AAC-caused reintubation patients were included in the case group in this study. Potential risk factors that had been noted in the investigation were classified into patient-specific, anesthetic-specific, operation-specific and outcome-related. Patient-specific factors included sex, age, body mass index (BMI), American Society of Anesthesiology (ASA) physical status, smoking history, sepsis, heart disease history, cerebral infarction history, asthma or chronic obstructive pulmonary disease (COPD) history, hypertension and diabetes mellitus. Anesthetic-specific factors included Cormack Lehane (CL) grade during intubation, anesthesiologist’s seniority, red blood cell (RBC) infusion, fresh frozen plasma (FFP) infusion, white blood cell (WBC) count, platelet count, red blood cell (RBC) count, alanine aminotransferase (ALT) level, albumin level and creatinine clearance rate (Ccr). Operative-specific factors included the surgical type, fluid load and duration of intubation. The outcome-related variables included postoperative hospital days and ICU days.

The definition of sepsis follows the Third International Consensus Definitions for Sepsis and Septic Shock (Sepsis-3) [6, 7]. Heart disease was defined as previous coronary artery disease, myocardial infarction, heart failure, structural cardiac disease or heart rhythm disease. Fluid load was defined as fluid total input minus total output divided by body weight. The duration of intubation was timed from the first successful intubation to the first completed extubation.

### Statistical analysis

Normally distributed continuous variables are expressed as the mean ± SD, and non-normally distributed continuous variables ware expressed as the median and interquartile range. Categorical variables are summarised as frequencies and percentages. Univariable analysis was used to compare the differences in potential risk factors between the cases and controls. Categorical variables were compared using chi-squared tests. Continuous variables were first tested for equality of variances using Levene’s test, and if the equal variance hypothesis was satisfied, a two-tailed independent t-test was used. The Mann-Whitney U test was used for non-normally distributed or unequal-variance data. Multivariable analysis was conducted to assess the associations between potential risk factors and AAC-caused unplanned reintubation using a multivariable logistic regression model. The variables chosen for the multivariable analysis were based on clinical experience and model fitting statistics. A two-sided *P*-value of < 0.05 was considered statistically significant. Statistical analyses were conducted using SPSS 19.0 (SPSS Inc. Chicago, IL, USA).

Because the sample size for the cases was identified and a matching ratio larger than 4 would lead to dramatically increased workload with no significant increase in the statistical power, we calculated the statistical power with the established maximum number of cases with a 1:4 matching ratio. The statistical power of this study ranged from 5.26 to 100.00% based on the potential risk factors. A power > 50% was achieved for 60.71% of the potential risk factors.

## Results

**Fig. 1:**
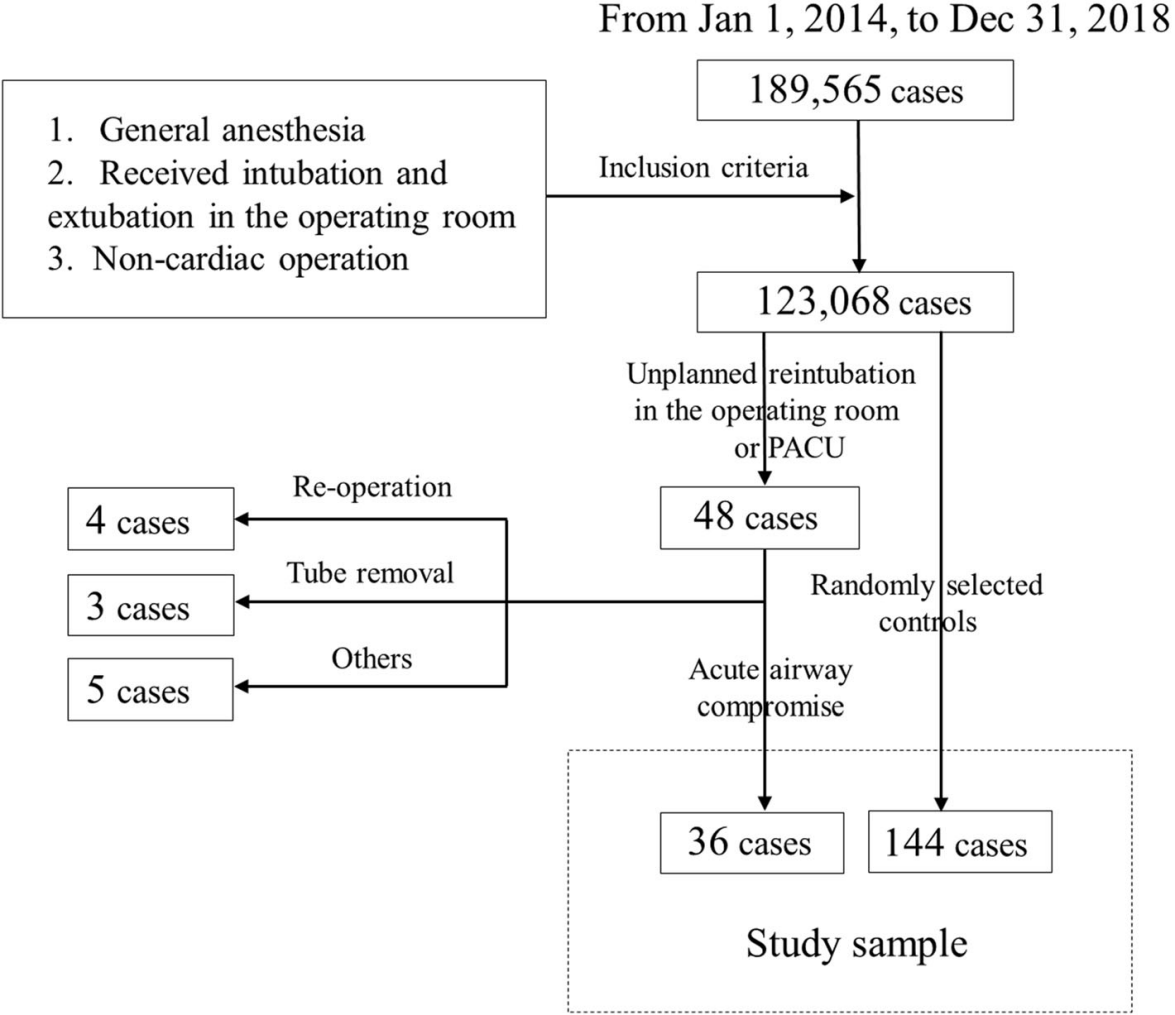
Flow chart of patient inclusion and exclusion

From 2014 to 2018, a total of 189,565 operations were performed in the PUMC Hospital operating room, and 123,068 cases qualified for inclusion according to our criteria (Fig. 1). Among these, 48 cases received unplanned reintubation in the operating room and PACU, and were categorised into four causes. Four cases were caused by emergent re-operative indications, such as unexpected excessive bleeding after extubation. Three cases were due to unexpected tube removal. Thirty-six cases were due to AAC and were enrolled in the case-control study. Five cases were due to other reasons, such as severe haemodynamic instability or neurological complications after a planned extubation. Specific causes of the 36 reintubation cases caused by AAC are shown in Table 1. Airway obstruction including laryngospasm/bronchospasm, excessive airway secretion, glossocoma, etc. was related to 17 cases. Five cases were possibly caused by respiratory muscle fatigue, for instance, residual muscular blockage. Four cases were associated with phrenic nerve dysfunction and were diagnosed with imaging examination. Five cases were possibly due to other reasons, such as adverse reactions to opioid drugs, acute respiratory distress syndrome (ARDS) or atelectasis. Reasons for five cases cannot be definitively identified.

**Table 1:** Possible causes for the 36 reintubation cases due to AAC

Possible causes	N (%)
**Airway obstruction**	17(47.22)
Laryngospasm/bronchospasm	11(30.56)
Excessive airway secretion	3(8.33)
Others	3(8.33)
**Respiratory muscle fatigue**	5(13.89)
Residual muscular blockage	3(8.33)
Others	2(5.56)
**Phrenic nerve dysfunction**	4(11.11)
**Others**	5(13.89)
**Unidentified reasons**	5(13.89)

*AAC* acute airway compromise

In this study, the incidence of overall unplanned reintubation was approximately 0.04%, and the incidence of reintubation caused by AAC was approximately 0.03%. All the cases and control groups underwent elective operations with intravenous and inhaled general anesthesia.

For the univariable analysis, the results of potential patient-specific risk factors showed that relative to the controls, sex, age, ASA physical status, sepsis, heart disease history and cerebral infarction history were highly associated with reintubation caused by AAC (Table 2). Statistical results of the potential anesthetic−/operative-specific risk factors and outcome-related variables revealed that patients with CL grade 3, high WBC counts, poor Ccrs, thoracic surgery, FFP infusion, increased fluid load and long intubation time were more likely to undergo AAC-caused reintubation (Table 3). Longer postoperative hospital and ICU stays were also associated with AAC-caused reintubation (Table 3).

**Table 2:** Patient-specific potential risk factors for unplanned reintubation caused by AAC

Variables	UR (*n* = 36)	Non-UR (*n* = 144)	OR/Mean difference (95%CI)	*P*-value
**Sex [n (%)]**				0.039^*^
Male	17(47.22)	42(29.17)	2.17(1.03 to 4.58)	
Female	19(52.78)	102(70.83)		
**Age (yr) [n (%)]**				
Mean	59.26 ± 19.25	46.07 ± 15.58		< 0.001^#^
> 65	17(47.22)	14(9.72)	8.33(3.53 to 19.61)	< 0.001^*^
≤ 65	19(52.78)	130(90.28)		
**BMI (kg m**^**−2**^)	24.71 ± 4.45	23.69 ± 3.84	1.01(−0.45 to 2.47)	0.172^#^
**ASA [n (%)]**				< 0.001^*^
1	5(13.89)	61(42.36)		
2	22(61.11)	77(53.47)	3.49(1.25 to 9.74)	0.017
3	9(25.00)	6(4.17)	18.30(4.61 to 72.58)	< 0.001
**Smoking history [n (%)]**				0.192^*^
Yes	10(27.78)	26(18.06)	1.75(0.75 to 4.07)	
No	26(72.22)	118(81.94)		
**Sepsis [n (%)]**				0.039^^^
Yes	2(5.56)	0(0.00)	–	
No	34(94.44)	144(100.00)		
**Heart disease history [n (%)]**				0.002^*^
Yes	9(25.00)	10(6.94)	4.46(1.66 to 12.05)	
No	27(75.00)	134(93.06)		
**Cerebral infarction history [n (%)]**				< 0.001^*^
Yes	8(22.22%)	2(1.39%)	20.41(4.08 to 100.00)	
No	28(77.78%)	142(98.61%)		
**Asthma/COPD history [n (%)]**				0.257^*^
Yes	2(5.56)	3(2.08)	2.76(0.44 to 17.24)	
No	34(94.44)	141(97.92)		
**Hypertension [n (%)]**				0.069^*^
Yes	13(36.11)	31(21.53)	2.06(0.94 to 4.52)	
No	23(63.89)	113(78.47)		
**Diabetes mellitus [n (%)]**				0.799^*^
Yes	3(8.33)	14(9.72)	0.84(0.23 to 3.11)	
No	33(91.67)	130(90.28)		

*AAC* acute airway compromise, *UR* unplanned reintubation, *OR* odds ratio, *CI* confidence interval, *BMI* body mass index

^*^Chi-square test

^#^Two-tailed independent t-test

^^^Fisher’s exact test, odds ratio cannot be estimated due to the zero event in the control group

**Table 3:** Anesthetic-/operative-specific potential risk factors and prognosis-related variables for unplanned reintubation caused by AAC

Variables	UR (*n* = 36)	Non-UR (*n* = 144)	OR/Mean difference (95%CI)	*P* value
**CL grade [n (%)]**				0.002^*^
1	19(52.78)	102(70.83)		
2	9(25.00)	36(25.00)	1.34(0.56 to 3.23)	0.512
3	6(16.67)	3(2.08)	10.74(2.47 to 46.69)	0.002
Laryngeal mask	2(5.56)	3(2.08)	3.58(0.56 to 22.88)	0.178
**Anesthesiologist seniority of (yr) [n (%)]**				0.879^*^
> 10	21(58.33)	86(59.72)	0.94(0.45 to 1.98)	
≤ 10	15(41.67)	58(40.28)		
**Surgical type [n (%)]**				< 0.001^*^
Head and neck	12(33.33)	40(27.78)	2.01(0.72 to 5.60)	0.180
Thoracic	12(33.33)	10(6.94)	8.06(2.54 to 25.58)	< 0.001
Laparoscopic	5(13.89)	47(32.64)	0.71(0.21 to 2.41)	0.588
Others	7(19.44)	47(32.64)		
**RBC infusion [n (%)]**				0.206^*^
Yes	3(8.33)	5(3.47)	2.53(0.57 to 1.11)	
No	33(91.67)	139(96.53)		
**FFP infusion [n (%)]**				< 0.001^*^
Yes	5(13.89)	1(0.69)	23.07(2.60 to 204.42)	
No	31(86.11)	143(99.31)		
**Fluid load (ml kg**^**−1**^**) [n (%)]**				0.009^*^
< 20	14(38.89)	96(66.67)		
20 ~ 40	18(50.00)	40(27.78)	3.09(1.40 to 6.80)	0.005
> 40	4(11.11)	8(5.55)	3.43(0.91 to 12.90)	0.068
**Intubation duration (min)(IQR)**	139.5(104.2, 266.0)	132.0(83.5, 189.5)	52.13(3.63 to 100.64)	0.049^+^
**WBC count (×10**^**9**^**/L)**	7.35 ± 2.80	6.26 ± 1.96	1.08(0.29 to 1.88)	0.008^#^
**PLT count (×10**^**9**^**/L)**	251.08 ± 80.21	231.87 ± 66.75	19.22(−6.38 to 44.81)	0.140^#^
**RBC count (× 10**^**12**^**/L)**	4.49 ± 0.59	4.47 ± 0.49	0.02(−0.17 to 0.20)	0.868^#^
**ALT (U/L)**	20.39 ± 12.30	23.13 ± 28.34	0.65(0.14 to 3.03)	0.573^#^
**ALB (g/L)**	42.08 ± 4.37	43.58 ± 4.57	−1.50(−3.16 to 0.17)	0.079^#^
**Ccr (ml/min) [n (%)]**				
Mean	81.03 ± 28.83	102.62 ± 29.04		< 0.001^#^
< 70	14(38.89)	14(9.72)	5.92(2.48 to 14.08)	< 0.001^*^
≥ 70	22(61.11)	130(90.28)		
**Postoperative hospital days (IQR)**	8.5(4.0 to 12.0)	4.0(2.0 to 7.0)	5.26(1.57 to 8.95)	< 0.001^+^
**Postoperative ICU days (IQR)**	1.0(0.0 to 5.0)	0.0(0.0 to 0.0)	3.94(1.69 to 6.18)	< 0.001^+^

*AAC* acute airway compromise, *UR* unplanned reintubation, *OR* odds ratio, *CI* confidence interval, *CL* Cormack Lehane, *RBC* red blood cell, *FFP* fresh frozen plasma, *IQR* interquartile range, *WBC* white blood cell, *PLT* platelets, *ALT* alanine aminotransferase, *ALB* albumin, *Ccr* creatinine clearance rate, *ICU* intensive care unit

^*^Chi-square test

^#^Two-tailed independent t-test

^+^Mann-Whitney U test

The multivariable analysis demonstrated that age > 65 yrs. (OR = 7.50, 95% CI 2.47–22.81, *P* < 0.001), ASA physical status 3 (OR = 6.51, 95% CI 1.18–35.92, *P* = 0.032), head-neck surgery (OR = 4.94, 95% CI 1.33–18.36, *P* = 0.017) or thoracic surgery (OR = 12.56, 95% CI 2.93–53.90, *P* < 0.001), and fluid load ≥20 ml kg^− 1^ (OR = 3.04, 95% CI 1.16–7.99, *P* = 0.024) were risk factors for reintubation due to AAC (Table 4).

**Table 4:** Multivariable regression of potential risk factors associated with unplanned reintubation caused by AAC

Variables	OR	95% CI	*P*-value
**Age > 65**	7.50	2.47 to 22.81	< 0.001
**Male sex**	1.95	0.74 to 5.15	0.178
**ASA Class**			
ASA1			
ASA2	1.77	0.56 to 5.54	0.331
ASA3	6.51	1.18 to 35.92	0.032
**Surgery type**			
Others			
Head and neck	4.94	1.33 to 18.36	0.017
Thoracic	12.56	2.93 to 53.90	< 0.001
Laparoscopic	1.69	0.39 to 7.25	0.480
**Fluid load≥20 ml kg**^−1^	3.04	1.16 to 7.99	0.024

*AAC* acute airway compromise, *OR* odds ratio, *CI* confidence interval, *ASA* American Society of Anesthesiology

**Fig. 2:**
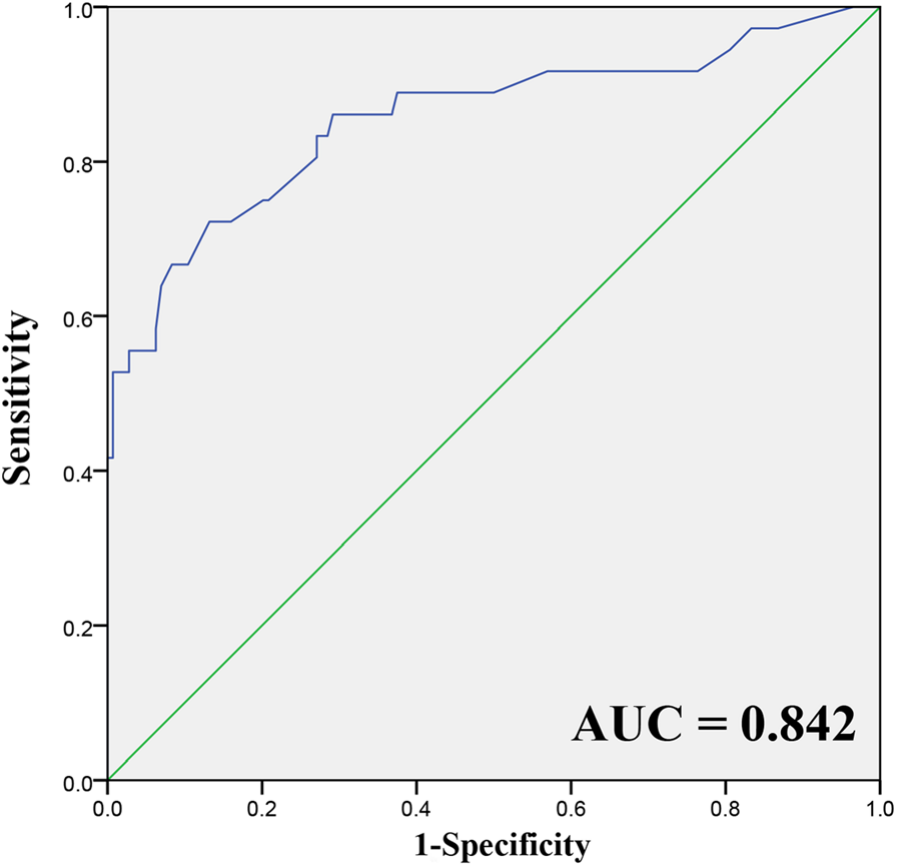
ROC curve of the logistic regression model. AUC = area under the curve

The receiver operating characteristic (ROC) curve was obtained using the logistic regression model (Fig. 2). The area under the curve (AUC) was 0.842 (95% CI 0.759–0.925), and the best cut-off point of this model was determined to correspond to a predicted probability of AAC-caused reintubation of 0.148, giving a specificity of 83.3% and a sensitivity of 73.6%.

## Discussion

Reintubation is required for various reasons [3]. Lin P H and colleagues reported that reintubation cases due to accidental removal or self-removal of the tracheal tube both had distinct risk factors and prognoses [8]. In this study, we classified the unplanned reintubation cases by cause, excluded the non-respiratory cases, and mainly focused on AAC-caused reintubation after general anesthesia since the postoperative respiratory compromise cases stood out as the majority (36/48, 75.0%).

Based on our results, age > 65 yrs. and ASA physical status 3 were identified as highly associated with unplanned reintubation caused by AAC. This result was in accordance with previous results that noted that advanced age and ASA physical status ≥3 should be considered independent risk factors for postoperative respiratory failure [9, 10]. Age ≥ 65 years was also shown to be an important risk factor for failed extubation in ICU patients [11]. For comorbidities, preoperative sepsis was found to have a positive association with reintubation due to AAC, which agrees with the previous literature [2, 12]. Heart disease history and cerebral infarction history were shown to be related to AAC-caused unplanned reintubation in the univariable analysis. Heart disease history is in line with previous results that found that patients with underlying chronic cardiac disease are at a high risk for extubation failure [1]. However, some studies reported no increased risk of reintubation in patients with comorbidities such as heart diseases [13], cerebrovascular accidents or central nervous system (CNS) diseases [14, 15]. For other comorbidities, a large-scale prospective study suggested that hypertension and insulin-required diabetes mellitus were independent predictors of unanticipated early postoperative intubation [2]. However, in this study, hypertension demonstrated a trend of increased risk in only the univariable analysis, although the result was not statistically significant. Stratifying patients by medication- or insulin-required diabetes mellitus may obtain more accurate results.

A multitude of studies have reported that COPD is highly associated with reintubation [1, 2, 14–17]. It was believed that COPD manifested as narrowing of the small airways, leading to an increase in breathing effort and exacerbating respiratory diaphragm muscle fatigue [18]. One possible reason that the results obtained from this study were not in accordance with previous reports is that in the PUMC Hospital, most moderate or severe COPD patients were sent back to the ICU straight after the operations and were thus excluded from the study.

To the best of our knowledge, no previous study has assessed the association between CL grade and reintubation. CL grading is usually used as a predictor for difficult intubation [19, 20]. In this study, CL grading was significant in the univariable analysis. We suspect that the crowded pharyngeal structure contributed to the collapsibility of the airway. Regarding laboratory results, a Ccr < 70 ml/min was demonstrated to be associated with AAC-caused unplanned reintubation in the univariable analysis. Numerous studies have concluded that chronic kidney disease [14] and renal insufficiency [2, 11, 17] are significant risk factors for reintubation. Although increased WBC counts were identified to be significant in the univariable analysis, they were more likely to be the result of confounding effects, rather than true associations. Hypoalbuminemia was also suggested to be highly associated with postoperative reintubation in some previous studies [11, 17]. However, such a result was not observed in this study.

A number of studies have reported the association between transfusion or RBC transfusion and reintubation [2]. To our knowledge, no previous study has assessed FFP transfusion as a potential risk factor for reintubation. Notably, we found FFP transfusion to be significant in the univariable analysis. Although reintubation patients could not be diagnosed with acute ARDS due to the absence of a blood-gas test, several studies have reported the relationship between FFP transfusion and ARDS [21]. Neto and colleagues found that perioperative FFP transfusion increased the risk of postoperative ARDS [22]. Thus, FFP transfusion may be correlated with ARDS and reintubation after operation. RBC transfusion was not identified as an independent risk factor, consistent with the result from Acheampong D and colleagues [11]. A fluid load ≥20 ml kg^− 1^ was revealed as a risk factor in this study; however, other studies found that fluid balance or overload was not a significant risk factor, possibly due to the different definitions of fluid load [15, 23]. There is evidence that an extensive infusion of fluid during an operation results in pulmonary edema and pneumonia [24], which may be correlated with unplanned reintubation.

For the operative-specific factors, head-neck surgery and thoracic surgery were identified as significant risk factors; this result is similar to those in previous reports [15, 16]. Of all the unplanned reintubated cases, four thoracic patients were ultrasonically diagnosed with phrenic nerve injury, which is an iatrogenic complication following thoracic and cardiac surgery, with an overall incidence ranging from 1 to 11% [25, 26]. In PUMC Hospital, all patients undergoing cardiac operations were extubated routinely in the ICU, therefore, all cardiac cases were excluded from the investigation.

In this study, the incidence rate of reintubation was low compared with that in the previous literature [13, 14]. There are mainly four feasible reasons for this variation in the results. First, all the anesthesiologists were trained by the same protocol despite seniority [27]. Second, extubation was routinely conducted by two anesthesiologists, and both the attending and resident doctors were responsible for the case. Third, most critical patients were sent back to the ICU after their operation, which also explained why there were no ASA physical status 4 or 5 patients in the study. Finally, although unplanned reintubation is mandatory to report in the adverse event reporting system, we suspect that there may have been some missing cases.

A small sample size was the major limitation for this study. As identified in the power estimate that was included in the statistical analysis, 60.71% of the potential risk factors had > 50% power, and the incidence of reintubation due to AAC was only 0.03%. This severely underpowered analysis may lead to false-negative results. Therefore, the potential factors that were found negative in this study will not necessarily be unrelated to the reintubation caused by AAC. Moreover, sepsis should be considered as an important risk factor for reintubation [2, 12], it also revealed statistical significance in the univariable analysis. However, due to the very limited sample size, only two patients were found in the case group while none was found in the control group. The zero event prevented us from including sepsis in the multivariable regression model. A larger sample size may be needed to verify these associations adequately. There were other limitations of this study. For instance, as this was a retrospective case-control study, there might be potential confounders that have causal associations with unplanned reintubation and are unbalanced between the case and control groups, therefore resulting in confounding effects. In addition, data on other potential risk factors, such as respiratory tract infection or hypothermia, which were considered significant in previous studies [15, 17], were not included in this study due to the limitation of the recording system. Prospective, multicenter studies with larger sample sizes and fewer untreated confounders are required to further validate the current conclusions.

One strength of this study was that it specified the cause of reintubation. Therefore, the evaluation of the risk factors had increased accuracy. Additionally, for the first time, we revealed that FFP transfusion and CL grades were significantly related to unplanned reintubation. Finally, all the cases and controls received a combination of intravenous and inhaled anesthesia; to some extent, we prevented the anesthetic method from acting as a confounding factor.

Here, we have attempted to identify risk factors for postoperative unplanned reintubation caused by AAC to prevent unplanned reintubation after general anesthesia. Thus, minimizing patient risk factors, staying alert and making judicious decisions are essential to improve surgical prognoses.

## Data Availability

The datasets generated and analyzed during the current study are available from the corresponding author on reasonable request.

## Abbreviations

AAC: Acute airway compromise
PUMC: Peking Union Medical College
ASA: American Society of Anesthesiology
BMI: Body mass index
COPD: Obstructive pulmonary disease
CL: Cormack Lehane
ALT: Alanine aminotransferase
WBC: White blood cell
RBC: Red blood cell
ALB: Albumin
Ccr: Creatinine clearance rate
PLT: Platelets
FFP: Fresh frozen plasma
ICU: Intensive care unit
ARDS: Acute respiratory distress syndrome
IQR: Interquartile range
